# Early Predictive Accuracy of Machine Learning for Hemorrhagic Transformation in Acute Ischemic Stroke: Systematic Review and Meta-Analysis

**DOI:** 10.2196/71654

**Published:** 2025-05-23

**Authors:** Benqiao Wang, Bohao Jiang, Dan Liu, Ruixia Zhu

**Affiliations:** 1 Department of Neurology First Hospital of China Medical University Shenyang China; 2 Department of Urology First Hospital of China Medical University Shenyang China

**Keywords:** machine learning, hemorrhagic transformation, acute ischemic stroke, predictive model, meta-analysis

## Abstract

**Background:**

Hemorrhagic transformation (HT) is commonly detected in acute ischemic stroke (AIS) and often leads to poor outcomes. Currently, there is no ideal tool for early prediction of HT risk. Recently, machine learning has gained traction in stroke management, prompting the exploration of predictive models for HT. However, systematic evidence on these models is lacking.

**Objective:**

In this study, we assessed the predictive capability of machine learning models for HT risk in AIS, aiming to inform the development of HT prediction tools.

**Methods:**

We conducted a thorough search of medical databases, such as Web of Science, Embase, Cochrane, and PubMed up until March 2025. The risk of bias was determined through the Prediction Model Risk of Bias Assessment Tool (PROBAST). Subgroup analysis was performed based on treatment backgrounds, diagnostic criteria, and types of HT.

**Results:**

A total of 83 eligible articles were included, containing 106 models and 88,197 patients with AIS with 9323 HT cases. There were 104 validation sets with a total c-index of 0.832 (95% CI 0.814-0.849), sensitivity of 0.82 (95% CI 0.79-0.84), and specificity of 0.78 (95% CI 0.74-0.81). Subgroup analysis indicated that the combined model achieved superior prediction accuracy. Moreover, we also analyzed the predictive performance of 6 mature models.

**Conclusions:**

Currently, although several prediction methods for HT have been developed, their predictive values are not satisfactory. Fortunately, our findings suggest that machine learning methods, particularly those combining clinical features and radiomics, hold promise for improving predictive accuracy. Our meta-analysis may provide evidence-based guidance for the subsequent development of more efficient clinical predictive models for HT.

**Trial Registration:**

PROSPERO CRD42024498997; https://www.crd.york.ac.uk/PROSPERO/view/CRD42024498997

## Introduction

Acute ischemic stroke (AIS) is a significant global health challenge with high disability and mortality rates worldwide [1]. It affects roughly 10.1 out of every million people globally and the case-fatality rate appears to be rising in recent years [2]. Fortunately, many patients are now benefiting from increasingly effective treatment methods. Positive and effective treatments, such as intravenous thrombolysis and mechanical thrombectomy, can improve functional outcomes, especially for cases within the limited time window [3]. However, some patients have poor outcomes in clinical practice, such as hemorrhagic transformation (HT), which increases the risk of mortality and disability in patients with AIS [4].

Several studies have demonstrated that matrix metalloproteinase-9 can independently predict HT in AIS [5,6]. Moreover, interleukin-33 was found to predict HT in AIS with an area under the curve of 0.739 [7]. In addition, serum S100B was reported to reflect the risk of HT in AIS after thrombolytic therapy [8]. However, the predictive performance of these markers remains contentious due to limitations such as small sample sizes, nonmulticenter study designs, and potential oversight of combined factors. Consequently, effective early prediction tools for HT are still lacking in clinical practice [9]. Therefore, it is essential to identify the HT risk early and guide the corresponding preventive measures.

Machine learning has been widely used in medicine in recent years. Multiple meta-analyses have shown that the models based on machine learning play an important role in the prognosis evaluation and treatment efficacy prediction of nervous system diseases, and machine learning has better prediction performance than clinical features [10-13]. In this setting, a series of models have been developed for early prediction of HT in patients with stroke based on machine learning. However, due to diversified methods of machine learning and various variables [14], there is a lack of systematic evidence on the predictive performance of these models, which is challenging for the subsequent development of simpler clinical prediction tools. Consequently, this systematic review and meta-analysis intended to explore the predictive performance of various prediction tools, including machine learning models, for HT in patients with AIS across different scenarios, to provide evidence-based guidance for promoting the development of artificial intelligence in this field and further developing simple and clinically practical prediction tools based on machine learning methods.

## Methods

### Registration of the Study

The study adhered to the PRISMA (Preferred Reporting Items for Systematic Reviews and Meta-Analyses) 2020 guidelines (Multimedia Appendix 1). Moreover, we have prospectively registered this study on PROSPERO (International Prospective Register of Systematic Reviews; CRD42024498997).

### Eligibility Criteria

The inclusion criteria were (1) patients with AIS; (2) the types of the included publications were case-cohort studies, nested case-control studies, cohort studies, or case-control studies; (3) a complete HT prediction model must be constructed; and (4) articles should be written in English.

Exclusion criteria were (1) types of research: meta-analyses, reviews, guidelines, publicly published conference abstracts without peer review and expert opinions; (2) studies that conducted only differential factor analysis without developing a complete machine learning model; (3) studies lacking outcome indicators for predictive accuracy of machine learning models, such as area under the curve, c-statistic, c-index, among others; or (4) publications with less than 20 cases.

### Data Retrieval and Screening

A systematic and comprehensive literature search was performed on the PubMed, Cochrane, Embase, and Web of Science databases from inception up to March 2025 using a combination of MeSH (Medical Subject Headings) terms, with no limitation on the year of publication or region. The general key terms used included brain infarct, stroke, machine learning, and intracerebral hemorrhage. Detailed search strategies are shown in Multimedia Appendix 2.

### Data Extraction and Analysis

The EndNote 20 (Clarivate) was used to filter the data and select eligible studies. Full-text versions of studies that met the inclusion criteria for this systematic review were then downloaded for further reading. Prior to data extraction, a structured spreadsheet was prepared, including fields such as title, first author, publication year, country, study type, patient source, treatment backgrounds, type of HT, diagnostic criteria for HT, follow-up duration, number of HTs, number of subjects, HT number of trainings, patient number of trainings, generation of validation set, methods to avoid overfitting, HT number of validation, patient number of validations, handling of missing values, variable selection, types of models, and variables for modeling. Subgroup analyses were performed according to different modeling variables (clinical features, radiomics, and radiomics + clinical features). Within modeling variables, subgroup analyses were also performed according to treatment background, diagnostic criteria, and type of HT.

A total of 2 investigators (BW and BJ) screened the identified studies and extracted data independently, and a third investigator (DL) was involved to cross-check the retrieved data.

### Risk of Bias in Studies

The risk of bias in the studies was reviewed using the Prediction Model Risk of Bias Assessment Tool (PROBAST) [15], which comprises specific questions divided into 4 domains: participants, predictor variables, outcome measures, and statistical methods. Each domain includes a set number of questions (2, 3, 6, and 9, respectively), with responses categorized as “yes or probably yes,” “no or probably no,” or “no information.” A domain was considered high risk if it included at least 1 “no or probably no” response. Conversely, a domain was deemed low risk if all responses were “yes or probably yes.” The overall risk of bias was characterized as low if all domains were rated low risk and high if at least 1 domain was rated high risk. The risk of bias was determined using PROBAST by 2 investigators (BW and BJ). The results were cross-verified by a third investigator (DL) in case of discrepancies. In addition, in the case where the above approach fails to resolve disagreements, the advice of blind review from another investigator (RZ) in our study, a professor with 20 years of experience in clinical practice and research will be ultimately adopted.

### Outcomes

The main outcome of our systematic review was the c-index, which indicates the overall accuracy of machine learning models. However, due to the potentially low incidence of HT in patients with AIS, some models may be developed using imbalanced data. Assessing machine learning accuracy solely based on the c-index presents challenges. Therefore, sensitivity and specificity were also considered our primary outcomes to provide a more comprehensive evaluation.

### Analysis Methods

A meta-analysis of the c-index was conducted to evaluate the accuracy of machine learning models. In some original studies, when the c-index lacked 95% CI and SE, we followed the study by Debray et al [16]. The statistical model was selected based on the heterogeneity index (*I*^2^). When *I*^2^>50%, the random-effects model was used; when *I*^2^<50%, the fixed-effects model was used. Considering the variations in model features among studies, a random-effects model was used to conduct a c-index meta-analysis. A bivariate mixed-effects model was used to conduct meta-analyses of specificity and sensitivity. Ideally, this analysis would use data from the diagnostic four-fold table, which was often missing in the original studies. Consequently, we used 2 complementary approaches to estimate the four-fold table: (1) combining the reported number of cases, precision, specificity, and sensitivity and (2) extracting specificity and sensitivity based on the optimal Youden Index and then calculating the number of cases. All meta-analyses were conducted using R (version 4.3.3; R development Core Team).

## Results

### Study Selection

**Figure 1:**
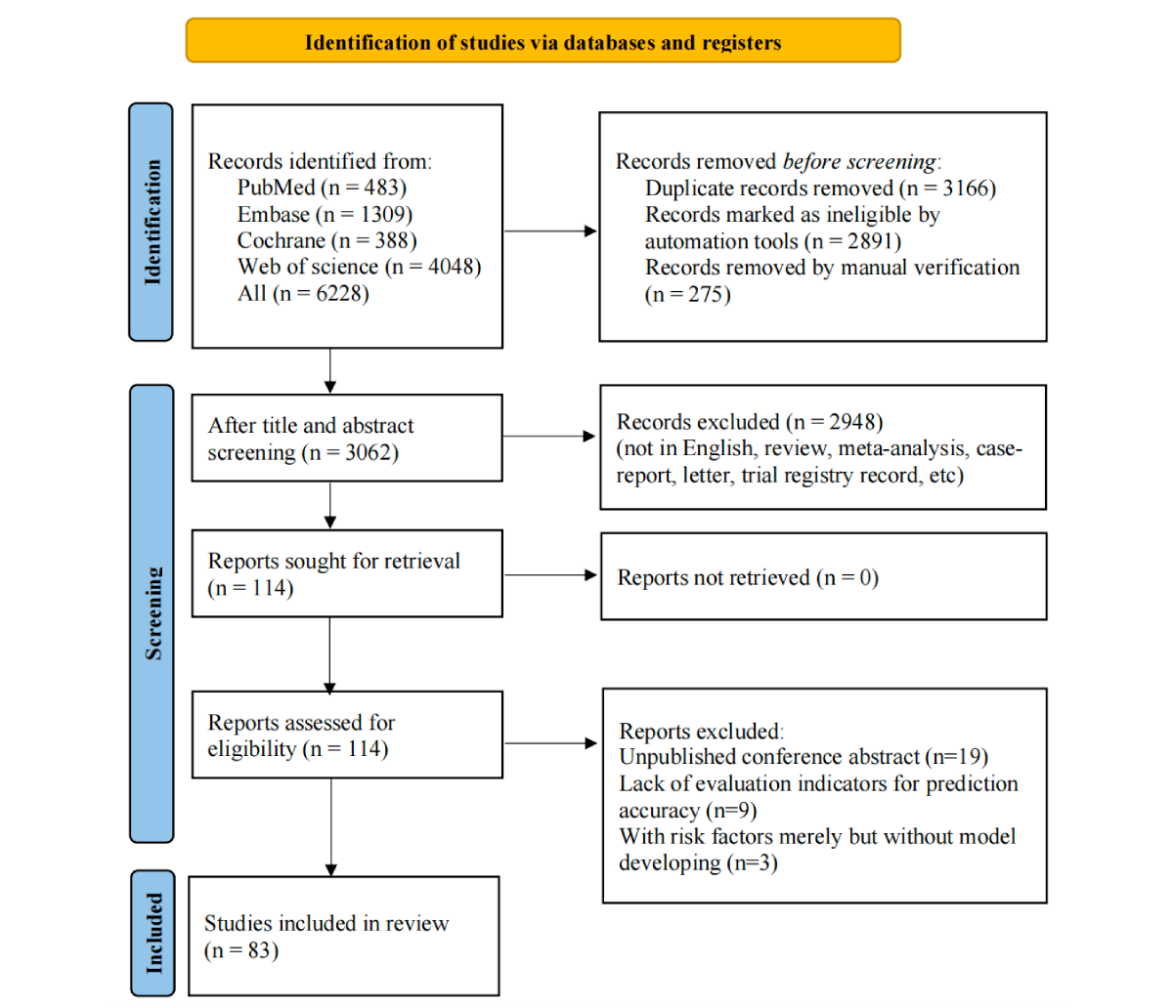
PRISMA flowchart of the literature selection process.

A systematic search for relevant articles was conducted across major medical databases including PubMed, Cochrane, Embase, and Web of Science. The search included publications up to March 2025. Following the initial retrieval of 6228 articles, we identified and removed 3166 duplicates. Subsequently, 2948 irrelevant publications were removed by browsing the title and abstract. Following a full-text review, 31 articles were removed due to the publicly published conference abstracts without peer review (n=19), lack of evaluation indicators for prediction accuracy (n=9), and absence of complete models (n=3). Finally, 83 articles [17-99] were included. The flow chart of the selection process is shown in Figure 1.

### Study Characteristics

All the eligible articles were published from 2002 to 2025. The original studies included in our study were mainly cohort studies (77 studies), with additional 4 case-control studies [32,36,72,76] and 2 studies with unclear study types [26,45]. A total of 106 models were developed using 5 types of modeling methods, mainly logistic regression and radiomics. Of the included studies, 102 articles developed new prediction models and 44 articles validated the mature models. Details about these studies, including their characteristics, are provided in Multimedia Appendix 3 [17-99].

### Quality Assessment

**Figure 2:**
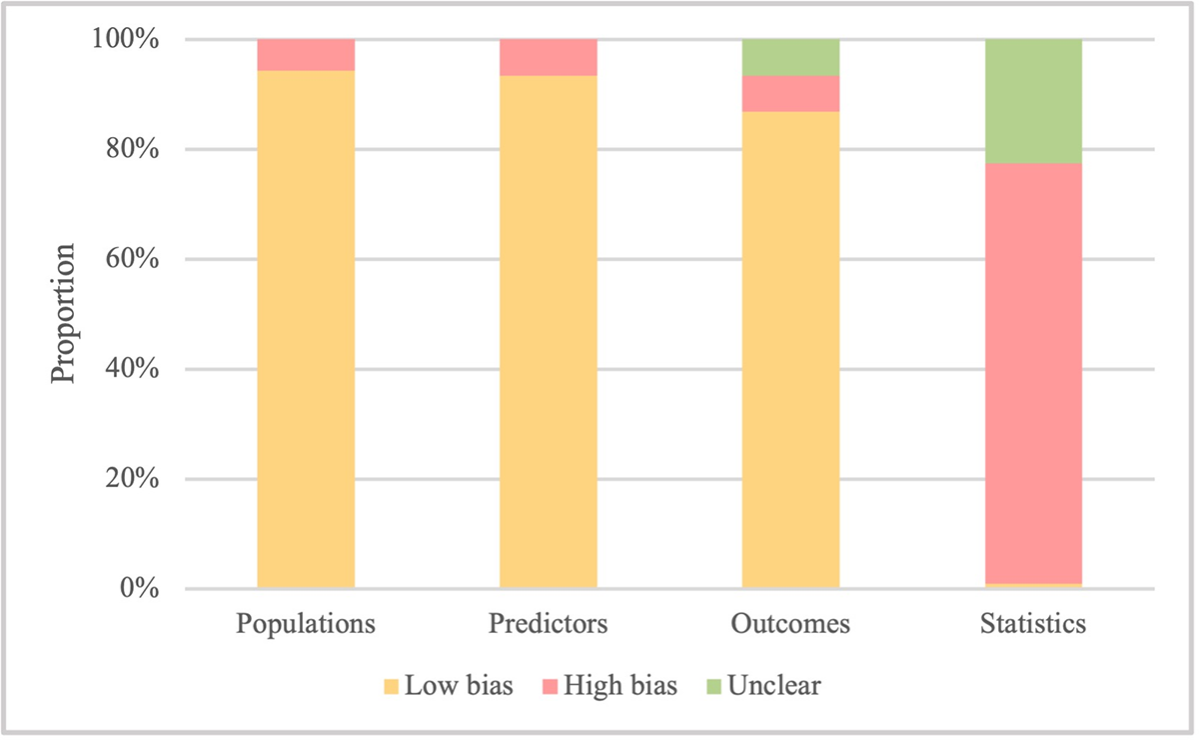
The risk-of-bias assessment of the included models.

The quality of studies was analyzed using the PROBAST checklists. The risk of bias was determined based on 4 dimensions, including populations, predictors, outcomes, and statistics. Notably, 6 models from 3 articles, which were case-control studies, were considered at risk of bias in the choice of subjects. The predictors of 7 models in 4 studies were selected on the premise of knowing the outcome, which increased the risk of bias in predictor selection and outcome ascertainment. Our analysis revealed that, overall, the statistical handling of data was the most common source of potential bias. Among the 19 models developed in the 19 articles, 14 models had an events per variable of less than 10, and 5 models had a sample size of less than 100. Our analysis identified several potential shortcomings in how some of the studies incorporated the data into their models. In 59 models from 42 articles, data with missing values were simply removed without any explanation. In addition, variables were selected based solely on univariate analysis in 1 model and 30 studies failed to consider the goodness of fit during development. All these issues can increase the risk of biased results. A detailed breakdown of these biases is presented in Figure 2 and Multimedia Appendix 4.

### The Results of Meta-Analysis

#### The Assessment of Predictive Value

This meta-analysis included 88,197 patients with AIS, among whom there were 9323 cases of HT. A total of 101 new machine learning models were developed using 101 training sets and 104 validation sets. All studies reported the c-index for predicting HT in patients with AIS. The overall c-index, calculated by a random-effects model, was 0.845 (95% CI 0.816-0.873) in the training sets. Furthermore, diagnostic fourfold tables were available from 81 models, showing an overall sensitivity of 0.84 (95% CI 0.81-0.86) and specificity of 0.81 (95% CI 0.79-0.84). In the validation sets, the pooled c-index was 0.832 (95% CI 0.814-0.849) in 104 sets and the overall sensitivity and specificity, obtained from 89 sets, were 0.82 (95% CI 0.79-0.84) and 0.78 (95% CI 0.74-0.81), specifically. The details of the analysis of newly developed models are presented in Tables 1 and 2. The c-index, sensitivity, and specificity of each included model are shown in Multimedia Appendix 5.

#### Subgroup Analysis

The newly developed models were classified into 3 categories based on their predictors: models that used only clinical features, models that used only radiomics, and models that combined clinical features with radiomics. In the training datasets, 64 models used clinical features, achieving an overall c-index of 0.819 (95% CI 0.788-0.851). Among these models, 48 included a diagnostic fourfold table, which indicated a pooled sensitivity of 0.81 (95% CI 0.79-0.84) and specificity of 0.79 (95% CI 0.75-0.83). Within the validation datasets, which consisted of 75 models, the overall c-index for models based on clinical features was 0.812 (95% CI 0.791-0.833). Moreover, the pooled sensitivity and specificity of 61 models were 0.81 (95% CI 0.78-0.84) and 0.74 (95% CI 0.69-0.79), respectively. Notably, 18 radiomics-based models in the training sets had an overall c-index of 0.909 (95% CI 0.885-0.932), with a sensitivity of 0.88 (95% CI 0.80-0.93) and specificity of 0.84 (95% CI 0.79-0.89) in 16 models of them. A total of 15 models in the validation sets had a pooled c-index of 0.880 (95% CI 0.842-0.918), sensitivity of 0.80 (95% CI 0.73-0.86), and specificity of 0.86 (95% CI 0.80-0.90).

**Figure 3:**
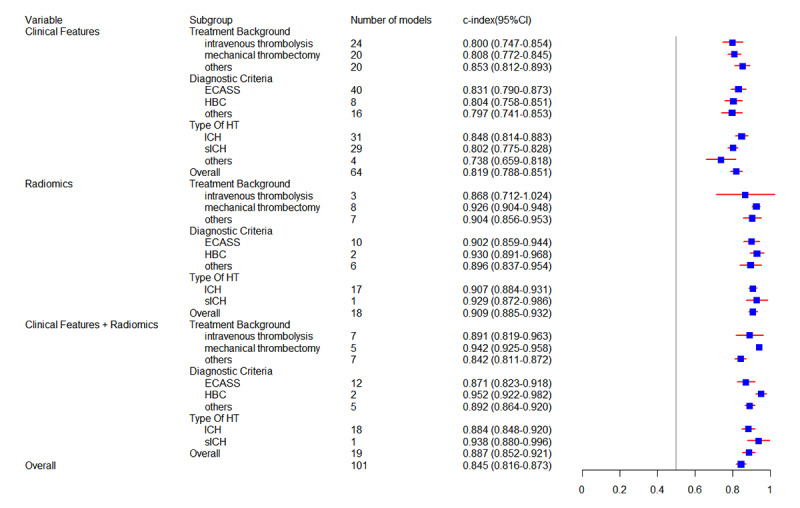
Forest plot of c-index for newly developed models in the training set. ECASS: European Cooperative Acute Stroke Study; HBC: Heidelberg Bleeding Classification; HT: hemorrhagic transformation; ICH: intracerebral hemorrhage; sICH: symptomatic intracerebral hemorrhage.

**Figure 4:**
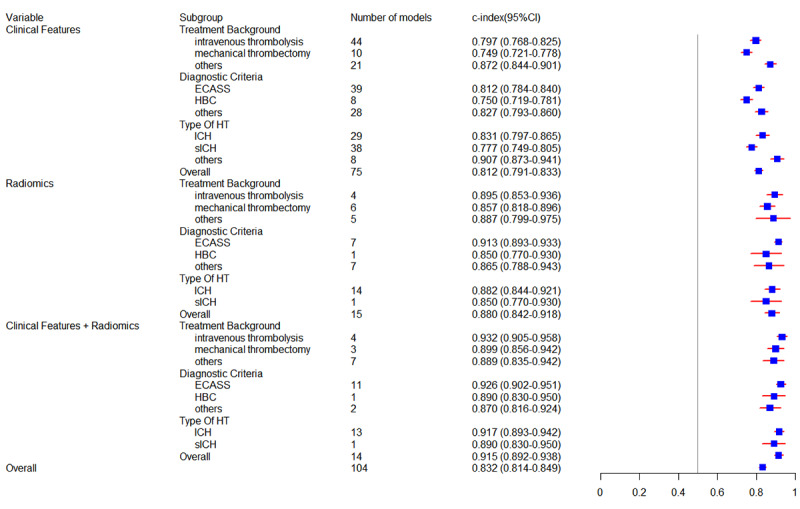
Forest plot of c-index for newly developed models in the validation set. ECASS: European Cooperative Acute Stroke Study; HBC: Heidelberg Bleeding Classification; HT: hemorrhagic transformation; ICH: intracerebral hemorrhage; sICH: symptomatic intracerebral hemorrhage.

In terms of models based on clinical features combined with radiomics, 19 models in the training sets and 14 models in the validation sets achieved an overall c-index of 0.887 (95% CI 0.852-0.921) and 0.915 (95% CI 0.892-0.938), respectively. Within this subgroup, 17 models in the training sets reported a pooled sensitivity of 0.86 (95% CI 0.81-0.89) and specificity of 0.84 (95% CI 0.79-0.88), while 13 models in the validation sets demonstrated an overall sensitivity of 0.86 (95% CI 0.82-0.89) and specificity of 0.81 (95% CI 0.76-0.86). Moreover, we analyzed the predictive performance of these newly developed models across different treatment backgrounds, diagnostic criteria, and types of HT within 3 subgroups. Detailed meta-analysis results of subgroup analysis are presented in Figures 3 and 4 and Tables 1 and 2.

**Table 1:** The c-index of newly developed models.

Subgroup analysis			Training set						Validation set									
			N^a^	Events	Sample size^b^	c-index (95% CI)	*I*²	N		Events	Sample size	c-index (95% CI)	*I*²					
**Clinical features**																		
	**Treatment background**																	
		Intravenous thrombolysis	24	1706	42,342	0.800 (0.747-0.854)	99.3	44		1971	21,536	0.797 (0.768-0.825)	95.6					
		Mechanical thrombectomy	20	1818	8674	0.808 (0.772-0.845)	93.3	10		478	3943	0.749 (0.721-0.778)	30					
		Others	20	1141	6812	0.853 (0.812-0.893)	93.9	21		420	3519	0.872 (0.844-0.901)	82.8					
	**Diagnostic criteria**																	
		ECASS^c^	40	2975	26,257	0.831 (0.790-0.873)	99.5	39		1366	14,422	0.812 (0.784-0.840)	92					
		HBC^d^	8	542	5100	0.804 (0.758-0.851)	88	8		373	3369	0.750 (0.719-0.781)	32.5					
		Others	16	941	25,081	0.797 (0.741-0.853)	95.4	28		1178	14,720	0.827 (0.793-0.860)	95.3					
	**Type of HT^e^**																	
		ICH^f^	31	1649	6761	0.848 (0.814-0.883)	96	29		1112	6314	0.831 (0.797-0.865)	93.2					
		sICH^g^	29	1734	35,305	0.802 (0.775-0.828)	92.9	38		1117	21,839	0.777 (0.749-0.805)	87.2					
		Others	4	1075	3732	0.738 (0.659-0.818)	95.6	8		773	2142	0.907 (0.873-0.941)	93.7					
Overall clinical features			64	4251	44,408	0.819 (0.788-0.851)	99.2	75		2812	28,044	0.812 (0.791-0.833)	94					
**Radiomics**																		
	**Treatment background**																	
		Intravenous thrombolysis	3	293	950	0.868 (0.712-1.024)	98	4		149	388	0.895 (0.853-0.936)	61.4					
		Mechanical thrombectomy	8	359	935	0.926 (0.904-0.948)	49.6	6		142	368	0.857 (0.818-0.896)	0.0					
		Others	7	345	746	0.904 (0.856-0.953)	91.2	5		176	334	0.887 (0.799-0.975)	85.1					
	**Diagnostic criteria**																	
		ECASS	10	590	1666	0.902 (0.859-0.944)	94.1	7		238	511	0.913 (0.893-0.933)	0.0					
		HBC	2	36	102	0.930 (0.891-0.968)	0.0	1		15	102	0.850 (0.770-0.930)	N/A^h^					
		Others	6	371	863	0.896 (0.837-0.954)	94.5	7		214	477	0.865 (0.788-0.943)	89.6					
	**Type of HT**																	
		ICH	17	997	2631	0.907 (0.884-0.931)	94.1	14		452	988	0.882 (0.844-0.921)	83.9					
		sICH	1	15	102	0.929 (0.872-0.986)	N/A	1		15	102	0.850 (0.770-0.930)	N/A					
Overall radiomics			18	997	2631	0.909 (0.885-0.932)	93.8	15		467	1090	0.880 (0.842-0.918)	83.8					
**Clinical features +radiomics**																		
	**Treatment background**																	
		Intravenous thrombolysis	7	382	1820	0.891 (0.819-0.963)	90.9	4		149	388	0.932 (0.905-0.958)	39.9					
		Mechanical thrombectomy	5	175	522	0.942 (0.925-0.958)	0.0	3		52	192	0.899 (0.856-0.942)	0.0					
		Others	7	158	326	0.842 (0.811-0.872)	34.1	7		115	220	0.889 (0.835-0.942)	59.4					
	**Diagnostic criteria**																	
		ECASS	12	528	2148	0.871 (0.823-0.918)	94	11		245	537	0.926 (0.902-0.951)	39.3					
		HBC	2	36	102	0.952 (0.922-0.982)	0.0	1		15	102	0.890 (0.830-0.950)	N/A					
		Others	5	151	418	0.892 (0.864-0.920)	0.0	2		56	161	0.870 (0.816-0.924)	0.0					
	**Type of HT**																	
		ICH	18	715	2668	0.884 (0.848-0.920)	92.4	13		301	698	0.917 (0.893-0.942)	44.2					
		sICH	1	15	102	0.938 (0.880-0.996)	N/A	1		15	102	0.890 (0.830-0.950)	N/A					
Overall clinical features+radiomics			19	715	2668	0.887 (0.852-0.921)	92	14		316	800	0.915 (0.892-0.938)	43.6					
Overall			101	5004	46,914	0.845 (0.816-0.873)	99.6	104		3082	28,697	0.832 (0.814-0.849)	93.3					

^a^N: the number of models.

^b^Sample size: total number of acute ischemic stroke cases included.

^c^ECASS: European Cooperative Acute Stroke Study.

^d^HBC: Heidelberg Bleeding Classification.

^e^HT: hemorrhagic transformation.

^f^ICH: intracerebral hemorrhage.

^g^sICH: symptomatic intracerebral hemorrhage.

^h^N/A: not applicable.

**Table 2:** The sensitivity and specificity of newly developed models.

Subgroup analysis^a^			Training set				Validation set		
			N^b^	Sen^c^ (95% CI)	Spe^d^ (95% CI)	N		Sen (95% CI)	Spe (95% CI)
**Clinical features**									
	**Treatment background**								
		Intravenous thrombolysis	13	0.84 (0.76-0.90)	0.82 (0.75-0.87)	33		0.78 (0.75-0.81)	0.80 (0.76-0.85)
		Mechanical thrombectomy	17	0.78 (0.73-0.82)	0.76 (0.72-0.80)	8		0.73 (0.67-0.79)	0.69 (0.62-0.75)
		Others	18	0.82 (0.80-0.84)	0.79 (0.71-0.86)	20		0.90 (0.84-0.94)	0.64 (0.49-0.76)
	**Diagnostic criteria**								
		ECASS^e^	33	0.83 (0.79-0.86)	0.81 (0.76-0.85)	35		0.82 (0.78-0.85)	0.66 (0.57-0.74)
		HBC^f^	6	0.79 (0.71-0.85)	0.70 (0.63-0.77)	6		0.76 (0.70-0.81)	0.65 (0.59-0.70)
		Others	9	0.78 (0.73-0.83)	0.79 (0.73-0.84)	20		0.83 (0.76-0.88)	0.86 (0.83-0.88)
	**Type of HT^g^**								
		ICH^h^	28	0.83 (0.79-0.86)	0.81 (0.76-0.86)	27		0.84 (0.80-0.87)	0.65 (0.53-0.76)
		sICH^i^	18	0.77 (0.73-0.81)	0.77 (0.73-0.81)	27		0.77 (0.73-0.80)	0.76 (0.72-0.80)
		Others	2	0.80-0.88	0.51-0.80	7		0.85 (0.71-0.93)	0.90 (0.85-0.94)
Overall clinical features			48	0.81 (0.79-0.84)	0.79 (0.75-0.83)	61		0.81 (0.78-0.84)	0.74 (0.69-0.79)
**Radiomics**									
	**Treatment background**								
		Intravenous thrombolysis	3	0.81-0.99	0.68-0.95	4		0.84 (0.74-0.91)	0.86 (0.76-0.92)
		Mechanical thrombectomy	8	0.88 (0.81-0.93)	0.85 (0.79-0.89)	6		0.71 (0.60-0.80)	0.87 (0.78-0.93)
		Others	5	0.76 (0.66-0.83)	0.85 (0.76-0.91)	5		0.85 (0.72-0.92)	0.85 (0.71-0.92)
	**Diagnostic criteria**								
		ECASS	10	0.90 (0.77-0.96)	0.86 (0.79-0.91)	7		0.82 (0.71-0.90)	0.89 (0.82-0.93)
		HBC	2	0.86-0.94	0.82-0.91	1		0.93	0.77
		Others	4	0.82 (0.76-0.86)	0.77 (0.66-0.85)	7		0.77 (0.66-0.85)	0.85 (0.73-0.92)
	**Type of HT**								
		ICH	15	0.87 (0.79-0.92)	0.84 (0.79-0.89)	14		0.80 (0.72-0.86)	0.87 (0.81-0.91)
		sICH	1	0.94	0.82	1		0.93	0.77
Overall radiomics			16	0.88 (0.80-0.93)	0.84 (0.79-0.89)	15		0.80 (0.73-0.86)	0.86 (0.80-0.90)
**Clinical features+radiomics**									
	**Treatment background**								
		Intravenous thrombolysis	6	0.92 (0.83-0.96)	0.85 (0.71-0.93)	4		0.86 (0.78-0.92)	0.87 (0.80-0.91)
		Mechanical thrombectomy	5	0.86 (0.82-0.90)	0.88 (0.86-0.90)	3		0.77-0.93	0.77-0.87
		Others	6	0.79 (0.75-0.82)	0.79 (0.74-0.83)	6		0.87 (0.82-0.90)	0.76 (0.68-0.82)
	**Diagnostic criteria**								
		ECASS	10	0.85 (0.77-0.90)	0.83 (0.75-0.89)	10		0.86 (0.82-0.90)	0.82 (0.75-0.88)
		HBC	2	0.89-0.93	0.82-0.89	1		0.93	0.77
		Others	5	0.86 (0.79-0.90)	0.84 (0.75-0.91)	2		0.77-0.82	0.79-0.81
	**Type of HT**								
		ICH	16	0.85 (0.81-0.89)	0.84 (0.79-0.89)	12		0.86 (0.82-0.89)	0.82 (0.76-0.87)
		sICH	1	0.93	0.82	1		0.93	0.77
Overall clinical features+radiomics			17	0.86 (0.81-0.89)	0.84 (0.79-0.88)	13		0.86 (0.82-0.89)	0.81 (0.76-0.86)
Overall			81	0.84 (0.81-0.86)	0.81 (0.79-0.84)	89		0.82 (0.79-0.84)	0.78 (0.74-0.81)

^a^For subgroups with fewer than 3 models, the range of sensitivity or specificity for that subgroup analysis is presented.

^b^N: the number of models.

^c^Sen: sensitivity.

^d^Spe: specificity.

^e^ECASS: European Cooperative Acute Stroke Study.

^f^HBC: Heidelberg Bleeding Classification.

^g^HT: hemorrhagic transformation.

^h^ICH: intracerebral hemorrhage.

^i^sICH: symptomatic intracerebral hemorrhage.

#### Validation of Mature Tools

**Figure 5:**
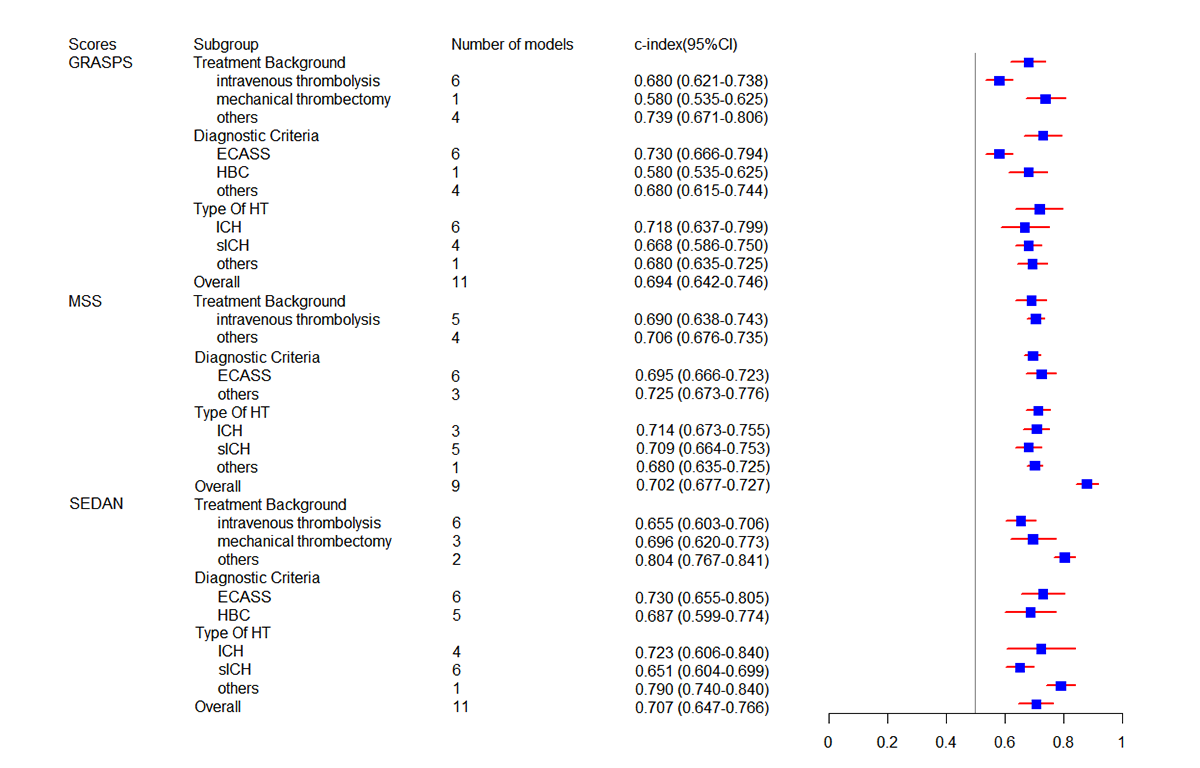
Forest plot of c-index for mature tools (GRASPS, MSS, and SEDAN). ECASS: European Cooperative Acute Stroke Study; HBC: Heidelberg Bleeding Classification; HT: hemorrhagic transformation; ICH: intracerebral hemorrhage; sICH: symptomatic intracerebral hemorrhage.

**Figure 6:**
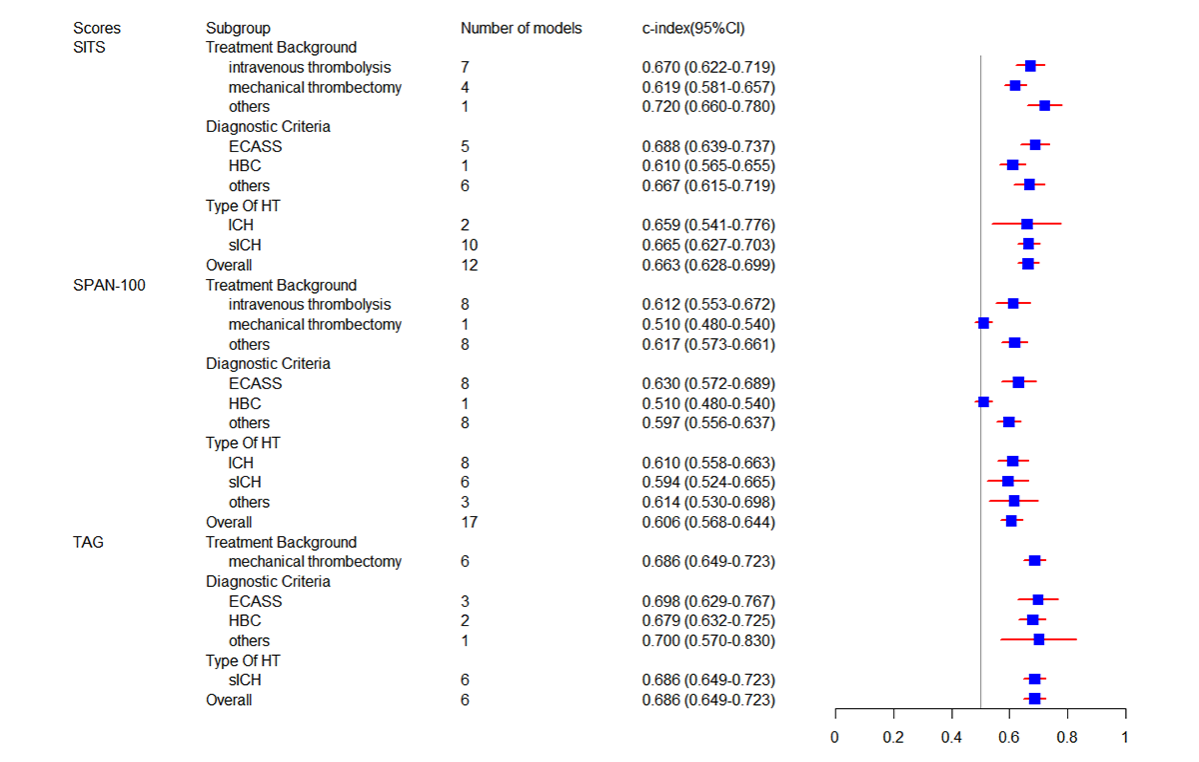
Forest plot of c-index for mature tools (SITS, SPAN-100, and TAG). ECASS: European Cooperative Acute Stroke Study; HBC: Heidelberg Bleeding Classification; HT: hemorrhagic transformation; ICH: intracerebral hemorrhage; sICH: symptomatic intracerebral hemorrhage.

In our study, we evaluated 6 established tools: GRASPS (Glucose, Race, Age, Sex, systolic blood pressure at presentation, and Severity of stroke at presentation), Multicenter rt-PA Stroke Survey score (MSS), SEDAN (baseline blood Sugar, Early infarct signs, hyper Dense cerebral artery sign, Age, NIH [National Institutes of Health] Stroke Scale), Safe Implementation of Treatments in Stroke (SITS), Stroke Prognostication using Age and NIH Stroke Scale (SPAN-100), and thrombolysis in cerebral ischaemia-Alberta stroke program early CT [computed tomography]-glucose score (TAG) [56,58,60,76,100,101]. A total of 11 datasets were used to validate the GRASPS, showing an overall c-index of 0.694 (95% CI 0.642-0.746), and the pooled sensitivity (0.71, 95% CI 0.60-0.80) and specificity (0.68, 95% CI 0.58-0.76) were calculated using 7 of these datasets. The overall c-index (0.702, 95% CI 0.677-0.727) of MSS was validated by 9 datasets, and its sensitivity (0.71, 95% CI 0.66-0.75) and specificity (0.67, 95% CI 0.64-0.71) were validated by 5 datasets. To validate SEDAN, the combined c-index across 11 datasets was 0.707 (95% CI 0.647-0.766), with sensitivity and specificity reported from 6 datasets as 0.75 (95% CI 0.65-0.82) and 0.69 (95% CI 0.58-0.77), respectively. In studies validating SITS, the pooled c-index across 12 datasets was 0.663 (95% CI 0.628-0.699). Sensitivity ranged from 0.4679 to 0.6159 and specificity ranged from 0.6957 to 0.6997 across 3 datasets. The overall c-index of 17 datasets to validate SPAN-100 was 0.606 (95% CI 0.568-0.644), and the sensitivity and specificity of 10 datasets were 0.42 (95% CI 0.26-0.59) and 0.86 (95% CI 0.76-0.93). Furthermore, from the 6 datasets which validated TAG, we could only obtain the pooled c-index of 0.686 (95% CI 0.649-0.723). Similarly, the predictive value of these mature tools under different backgrounds of treatments, diagnostic criteria, and HT types was also explored. Detailed meta-analysis results for these mature models are presented in Figures 5 and 6 and Tables 3 and 4.

**Table 3:** The c-index of mature models.

Subgroup analysis				Validation set						
				N^a^		Events^b^		Sample size^c^		c-index (95% CI)
**GRASPS^d^**										
	**Treatment background**									
		Intravenous thrombolysis	6		276		2112		0.680 (0.621-0.738)	
		Mechanical thrombectomy	1		188		3180		0.580 (0.535-0.625)	
		Others	4		433		2132		0.739 (0.671-0.806)	
	**Diagnostic criteria**									
		ECASS^e^	6		346		2879		0.730 (0.666-0.794)	
		HBC^f^	1		188		3180		0.580 (0.535-0.625)	
		Others	4		363		3249		0.680 (0.615-0.744)	
	**Type of HT^g^**									
		ICH^h^	6		249		2360		0.718 (0.637-0.799)	
		sICH^i^	4		435		6429		0.668 (0.586-0.750)	
		Others	1		213		1884		0.680 (0.635-0.725)	
Overall GRASPS				11		897		7424		0.694 (0.642-0.746)
**MSS** ^j^										
	**Treatment background**									
		Intravenous thrombolysis	5		171		642		0.690 (0.638-0.743)	
		Others	4		433		2132		0.706 (0.676-0.735)	
	**Diagnostic criteria**									
		ECASS	6		374		2774		0.695 (0.666-0.723)	
		Others	3		230		1973		0.725 (0.673-0.776)	
	**Type of HT**									
		ICH	3		111		801		0.714 (0.673-0.755)	
		sICH	5		280		1973		0.709 (0.664-0.753)	
		Others	1		213		1884		0.680 (0.635-0.725)	
Overall MSS				9		604		2774		0.702 (0.677-0.727)
**SEDAN^k^**										
	**Treatment background**									
		Intravenous thrombolysis	6		345		1648		0.655 (0.603-0.706)	
		Mechanical thrombectomy	3		51		258		0.696 (0.620-0.773)	
		Others	2		151		787		0.804 (0.767-0.841)	
	**Diagnostic criteria**									
		ECASS	6		171		789		0.730 (0.655-0.805)	
		Others	5		376		2251		0.687 (0.599-0.774)	
	**Type of HT**									
		ICH	4		198		1807		0.723 (0.606-0.840)	
		sICH	6		258		1712		0.651 (0.604-0.699)	
		Others	1		91		539		0.790 (0.740-0.840)	
Overall SEDAN				11		547		2693		0.707 (0.647-0.766)
**SITS^l^**										
	**Treatment background**									
		Intravenous thrombolysis	7		407		1994		0.670 (0.622-0.719)	
		Mechanical thrombectomy	4		239		3438		0.619 (0.581-0.657)	
		Others	1		60		248		0.720 (0.660-0.780)	
	**Diagnostic criteria**									
		ECASS	5		178		1143		0.688 (0.639-0.737)	
		HBC	1		188		3180		0.610 (0.565-0.655)	
		Others	6		340		2252		0.667 (0.615-0.719)	
	**Type of HT**									
		ICH	2		174		1605		0.659 (0.541-0.776)	
		sICH	10		532		5432		0.665 (0.627-0.703)	
Overall SITS				12		706		5680		0.663 (0.628-0.699)
**SPAN-100^m^**										
	**Treatment background**									
		Intravenous thrombolysis	8		428		2343		0.612 (0.553-0.672)	
		Mechanical thrombectomy	1		188		3180		0.510 (0.480-0.540)	
		Others	8		682		3389		0.617 (0.573-0.661)	
	**Diagnostic criteria**									
		ECASS	8		502		3241		0.630 (0.572-0.689)	
		HBC	1		188		3180		0.510 (0.480-0.540)	
		Others	8		608		4464		0.597 (0.556-0.637)	
	**Type of HT**									
		ICH	8		387		2975		0.610 (0.558-0.663)	
		sICH	6		570		6660		0.594 (0.524-0.665)	
		Others	3		341		2668		0.614 (0.530-0.698)	
Overall SPAN-100				17		1298		8912		0.606 (0.568-0.644)
**TAG^n^**										
	**Treatment background**									
		Mechanical thrombectomy	6		209		1499		0.686 (0.649-0.723)	
	**Diagnostic criteria**									
		ECASS	3		56		367		0.698 (0.629-0.767)	
		HBC	2		141		1132		0.679 (0.632-0.725)	
		Others	1		12		258		0.700 (0.570-0.830)	
	**Type of HT**									
		sICH	6		209		1499		0.686 (0.649-0.723)	
Overall TAG				6		209		1499		0.686 (0.649-0.723)

^a^N: the number of models.

^b^Events, patients with hemorrhagic transformation.

^c^Sample size: total number of acute ischemic stroke cases included.

^d^GRASPS: Glucose, Race, Age, Sex, systolic blood Pressure at presentation, and Severity of stroke at presentation.

^e^ECASS: European Cooperative Acute Stroke Study.

^f^HBC: Heidelberg Bleeding Classification.

^g^HT: hemorrhagic transformation.

^h^ICH: intracerebral hemorrhage.

^i^sICH: symptomatic intracerebral hemorrhage.

^j^MSS: Multicenter rt-PA Stroke Survey score.

^k^SEDAN: baseline blood Sugar, Early infarct signs, hyper-Dense cerebral artery sign, Age, NIH (National Institutes of Health) Stroke Scale.

^l^SITS: Safe Implementation of Treatments in Stroke.

^m^SPAN-100: Stroke Prognostication using Age and NIH Stroke Scale.

^n^TAG: thrombolysis in cerebral ischemia-Alberta stroke program early CT [computed tomography]-glucose score.

**Table 4:** The sensitivity and specificity of mature models.

Subgroup analysis					Validation set				
					N^a^		Sen^b^ (95% CI)		Spe^c^ (95% CI)
**GRASPS^d^**									
	**Treatment background**								
		Intravenous thrombolysis		4		0.77 (0.50-0.92)		0.67 (0.49-0.80)	
		Others		3		0.67-0.76^e^		0.62-0.75^e^	
	**Diagnostic criteria**								
		ECASS^f^		5		0.67 (0.55-0.77)		0.65 (0.52-0.77)	
		Others		2		0.70-0.76^e^		0.68-0.75^e^	
	**Type of HT^g^**								
		ICH^h^		4		0.77 (0.50-0.92)		0.67 (0.49-0.80)	
		sICH^i^		2		0.70-0.76^e^		0.68-0.75^e^	
		Others		1		0.67^j^		0.62^j^	
Overall GRASPS					7		0.71 (0.60-0.80)		0.68 (0.58-0.76)
**MSS^k^**									
	**Treatment background**								
		Intravenous thrombolysis		2		0.65-0.77^e^		0.63-0.68^e^	
		Others		3		0.68-0.76^e^		0.63-0.73^e^	
	**Diagnostic criteria**								
		ECASS		3		0.65-0.77^e^		0.63-0.68^e^	
		Others		2		0.68-0.76^e^		0.68-0.73^e^	
	**Type of HT**								
		ICH		2		0.65-0.77^e^		0.63-0.68^e^	
		sICH		2		0.68-0.76^e^		0.68-0.73^e^	
		Others		1		0.69^j^		0.63^j^	
Overall MSS					5		0.71 (0.66-0.75)		0.67 (0.64-0.71)
**SEDAN^l^**									
	**Treatment background**								
		Intravenous thrombolysis		2		0.50-0.75^e^		0.68-0.73^e^	
		Mechanical thrombectomy		3		0.62-0.89^e^		0.41-0.73^e^	
		Others		1		0.71^j^		0.78^j^	
	**Diagnostic criteria**								
		ECASS		4		0.74 (0.56-0.86)		0.64 (0.50-0.76)	
		Others		2		0.67-0.71^e^		0.73-0.78^e^	
	**Type of HT**								
		ICH		2		0.50-0.75^e^		0.67-0.74^e^	
		sICH		3		0.62-0.89^e^		0.41-0.73^e^	
		Others		1		0.71^j^		0.78^j^	
Overall SEDAN					6		0.75 (0.65-0.82)		0.69 (0.58-0.77)
**SITS^m^**									
	**Treatment background**								
		Mechanical thrombectomy		3		0.47-0.62^e^		0.69-0.70^e^	
	**Diagnostic criteria**								
		ECASS		2		0.47-0.62^e^		0.69-0.70^e^	
		Others		1		0.58^j^		0.70^j^	
	**Type of HT**								
		sICH		3		0.47-0.62^e^		0.69-0.70^e^	
Overall SITS					3		0.47-0.62^e^		0.69-0.70^e^
**SPAN-100^n^**									
	**Treatment background**								
		Intravenous thrombolysis		4		0.50 (0.20-0.80)		0.86 (0.72-0.94)	
		Others		6		0.41 (0.21-0.63)		0.87 (0.71-0.95)	
	**Diagnostic criteria**								
		ECASS		5		0.46 (0.27-0.66)		0.82 (0.67-0.92)	
		Others		5		0.37 (0.16-0.63)		0.89 (0.74-0.96)	
	**Type of HT**								
		ICH		5		0.44 (0.19-0.72)		0.89 (0.78-0.95)	
		sICH		2		0.65-0.76^j^		0.63-0.70^j^	
		Others		3		0.15-0.65^e^		0.65-0.98^e^	
Overall SPAN-100					10		0.42 (0.26-0.59)		0.86 (0.76-0.93)
**TAG^o^**									
	**Treatment background**								
			Mechanical thrombectomy	4		0.78 (0.62-0.89)^e^		0.55 (0.46-0.64)	
	**Diagnostic criteria**								
			ECASS	3		0.71-0.93^e^		0.40-0.66^e^	
			Others	1		0.75^j^		0.57^j^	
	**Type of HT**								
			sICH	4		0.78 (0.62-0.89)		0.55 (0.46-0.64)	
Overall TAG					4		0.78 (0.62-0.89)		0.55 (0.46-0.64)

^a^N, the number of models.

^b^Sen, sensitivity.

^c^Spe, specificity.

^d^GRASPS: Glucose, Race, Age, Sex, systolic blood Pressure at presentation, and Severity of stroke at presentation.

^e^Subgroups with less than 3 models are expressed as intervals of sensitivity and specificity within that group.

^f^ECASS: European Cooperative Acute Stroke Study.

^g^HT: hemorrhagic transformation.

^h^ICH: intracerebral hemorrhage.

^i^sICH: symptomatic intracerebral hemorrhage.

^j^Only one value indicates the corresponding sensitivity or specificity, since only one model was involved in this subgroup.

^k^MSS: Multicenter rt-PA Stroke Survey score.

^l^SEDAN: baseline blood Sugar, Early infarct signs, hyper-Dense cerebral artery sign, Age, NIH (National Institutes of Health) Stroke Scale.

^m^SITS: Safe Implementation of Treatments in Stroke.

^n^SPAN-100: Stroke Prognostication using Age and NIH Stroke Scale.

^o^TAG: thrombolysis in cerebral ischaemia-Alberta stroke program early CT [computed tomography]-glucose score.

## Discussion

### Principal Findings

Our systematic review and meta-analysis identified several existing tools for predicting HT risk in patients with AIS. However, the predictive performance of these currently available tools remains controversial. In recent studies, some machine learning models were built mainly based on clinical features, radiomics, and clinical features combined with radiomics, which similarly had a relatively ideal predictive value. The validation sets yielded c-index values of 0.812 (95% CI 0.791-0.833) for models based on clinical features, 0.880 (95% CI 0.842-0.918) for models based on radiomics, and 0.915 (95% CI 0.892-0.938) for models integrating clinical features with radiomics. These findings suggest that the models based on both clinical features and radiomics demonstrated superior predictive accuracy to those using either clinical features or radiomics alone. This study provided evidence-based evidence for the use of machine learning to develop models based on different modeling variables (clinical features, radiomics, and radiomics combined with clinical features). In the future, with the help of the well-developed prediction model, the high-risk population of HT can be screened, according to which, doctors can take active treatment and intervention, and increase the density and attention of follow-up to reduce the risk of HT of patients with AIS.

### Subgroup Evaluation

In addition to summarizing the overall accuracy of machine learning models for HT prediction, we also conducted subgroup analyses to explore potential variations in model performance. Notably, Tan et al [102] performed a separate meta-analysis on 21 studies encompassing 4473 patients with AIS. Their analysis focused on comparing the efficacy and safety of endovascular treatment and intravenous thrombolysis, finding no significant difference in HT risk between the 2 methods. In our study, detailed subgroup analyses were also performed by different treatment backgrounds. The c-index of these models for predicting HT after thrombolysis and thrombectomy was similar, while the models for predicting thrombolysis showed better sensitivity and specificity. However, we cannot rule out the influence of the fewer models for predicting thrombectomy. Overall, under the contexts of all these treatment backgrounds, machine learning models can be established to predict HT.

Currently, the diagnostic criteria for HT primarily encompass the European Cooperative Acute Stroke Study (ECASS) [67,103,104] and Heidelberg Bleeding Classification (HBC) [105]. ECASS defines symptomatic intracerebral hemorrhage (sICH) strictly, focusing on significant increases in the National Institutes of Health Stroke Scale (NIHSS) score (≥4) from baseline and neurological deterioration. However, the definition of sICH in HBC is broader and more flexible, and sICH can be identified when any of the following criteria are satisfied: a NIHSS score exceeding baseline value (≥4), an increase in 1 NIHSS category (≥2), requiring a major medical or surgical intervention or disease progression without known causes. In contrast, asymptomatic intracerebral hemorrhage and other types of hemorrhage are mainly defined based on imaging. Among the included newly developed models, out of the 27 models for thrombolysis, 17 models adopted ECASS criteria, whereas among the 21 models for thrombectomy, 11 adopted ECASS criteria, and 8 adopted HBC criteria. In this study, we found that the ECASS models had higher predictive accuracy for HT compared to the model based on HBC. However, this result is concluded only based on limited evidence. To assess its generalizability across different hemorrhage subtypes, we conducted a subgroup analysis stratified by the type of HT. The HT categories included sICH, intracerebral hemorrhage without further subtype classification, and other types of intracerebral hemorrhage distinct from sICH. This analysis revealed that the machine learning models maintained relatively stable predictive performance across the various HT subtypes. Our analysis included studies that evaluated clinical feature–based models, radiomics-based models, and combination models. All model types demonstrated a degree of predictive ability. Notably, clinical feature-based models offer clear advantages in interpretability due to the use of well-defined variables. However, the meta-analysis revealed the lowest pooled c-index (0.812 in the validation set) for models based solely on interpretable clinical features. Conversely, radiomics-based models exhibited improved predictive accuracy, reflected by an overall c-index of 0.880. Notably, the segmentation of medical imaging, feature extraction, and difference of imaging time points can potentially cause heterogeneity during model establishment. In addition, the type of medical imaging selected can significantly impact the model’s results. These limitations underscore the need for further research to solidify the predictive power of radiomics models. Our analysis revealed that models based on both clinical features and radiomics achieved the best overall performance (c-index of 0.915 in the validation set), suggesting their potential utility in future clinical practice.

### Comparison With Other Studies

Previous investigations have focused on the early prediction of HT in patients with AIS. In a meta-analysis by Suh et al [106] assessing perfusion CT for predicting HT risk in AIS, they reported a c-index of 0.84 (95% CI 0.81-0.87), sensitivity of 0.84 (95% CI 0.71-0.91), and specificity of 0.74 (95% CI 0.67-0.81). This meta-analysis comprised 15 original studies that reported CT perfusion parameters. The results showed that high blood-brain barrier permeability and low perfusion were associated with HT. Moreover, another meta-analysis of 9 studies obtained a c-index of models based on magnetic resonance imaging parameters of 0.85 (95% CI 0.82-0.88), sensitivity of 0.92 (95% CI 0.70-0.98), and specificity of 0.78 (95% CI 0.65-0.87) to predict the risk of HT [107]. Increased permeability, hypoperfusion, reduced apparent diffusion coefficient values, and fluid-attenuated inversion recovery hyperintensity are known to elevate the risk of HT. Interestingly, radiomics leverages high-throughput computing to extract numerous quantitative image-derived features, thereby enhancing diagnostic, prognostic, and predictive accuracy [108].

Furthermore, clinical features have been used to develop accurate prediction models. Interpretable clinical features of HT were analyzed in a previous study [109], with the c-index ranging from 0.67 to 0.79 in the included original studies and the c-index ranging from 0.59 to 0.88 in the external validation studies. The present results indicated that the prediction models based on these common clinical features possess promising predictive value.

Although all of the previously developed models have certain effects on HT prediction, the predictive value of the models based on either conventional imaging parameters or clinical features is not as good as that based on machine learning. Our study shows that the c-index of the prediction models built based on clinical features and radiomics can even reach 0.812 and 0.880 in the validation set, respectively, which largely demonstrates the potential of prediction models based on machine learning to be applied in clinical practice. In addition, the prediction model developed by clinical features combined with radiomic features had the best predictive value with a c-index of 0.915, which provides an important reference for the development of subsequent HT prediction models and is in line with our conventional knowledge. Since radiomics only considers the characteristics of the lesion itself and demographics, which are part of the clinical characteristics, can also influence the risk of developing HT [110,111], only a combination of the two can predict HT more comprehensively. The predictive efficacy of radiomics is affected by various factors, and there is currently no uniform specification and supervision for the establishment of predictive models based on radiomics [112], which poses a challenge to its clinical application. Overall, compared with other studies, our study provides stronger references for subsequent HT prediction models.

### Strengths and Limitations

This is the first systematic review encompassing all types of machine learning–based predictive models for HT in patients with AIS. In addition, robust subgroup analysis was conducted by different treatment backgrounds, diagnostic criteria, and HT types. Moreover, we analyzed the external validation results of mature models and found that the machine learning models had good performance, indicating they are promising tools for the establishment of new HT prediction models in the future.

The limitations of this analysis need to be acknowledged. First, the number of included models did not allow us to directly compare the performance of various machine learning algorithms using the same set of model features. Second, most original studies validated their models internally rather than externally, thus probably impacting the interpretation of the results. Our detailed subgroup analysis also resulted in too few externally validated models in each subgroup. Moreover, radiomics-based studies may be more dependent on imaging parameters and the experience of the segmenter. Therefore, future studies should incorporate patients from multicenter and different population backgrounds to construct models with wider applicability and higher robustness under standard operation processes for accurate treatment and prevention. Third, treatment backgrounds were diverse among the involved models. Despite the comprehensive discussion of treatment contexts, there is a large variation in the number of cases included in different treatment contexts (thrombolysis and thrombectomy) and the number of models. Hence, these results still need to be interpreted with caution. Fourth, the limitation of our research also lies in the lack of stratified analysis of geographical and demographic differences. In addition, for some studies with too small a sample size, prediction models developed using deep learning cannot completely avoid the risk of overfitting. Moreover, publication bias was not assessed in our study because publication bias is more inclined to be assessed in the same model. Finally, fewer studies validated existing mature models, and, some of the tools were not designed to predict HT, such as SITS, which was built to predict the prognosis of patients with stroke [101]. Therefore, these results should be interpreted with caution.

### Future Prospects

Nowadays, although machine learning has a wide range of applications in the research of AIS, it still faces certain challenges for its promotion in clinical practice. First, in the process of machine learning development, a large number of models tend to be developed based on a small number of cases, which raises some concerns about the robustness of the models. In the screening process of variables, the process of obtaining some variables is not clear, especially the findings based on demographic information that involves private information, which may compromise the accuracy of the variables. In the process of model construction, particularly for models based on radiomics, there is a lack of standards for the process of image selection, image segmentation, and texture extraction and screening, which makes model development heavily dependent on experience. In addition, existing models are predominantly internally validated and only a few studies have carried out external validation in different regions, which poses a challenge to model promotion and accuracy. Especially in radiomics-based models, there may be large differences in images from different institutions and there is still a lack of standardized criteria in clinical practice. Moreover, a large number of existing models only consider the accuracy of the model without considering risk stratification of the population, especially the variability of health care resources in different regions, which also poses a challenge to the clinical application of the model. In addition, the ethical issues involved in models developed based on machine learning are also worth noting. Although the prediction model has an ideal application prospect, it is after all a means based on artificial intelligence. In the future, clinical diagnosis and treatment can not completely rely on the model established by machine learning. We should also pay attention to humanistic care and personalized diagnosis and treatment in clinical practice, especially for patients identified as low-risk by predictive models, we should not ignore the systematic management of them. In summary, in the future, we should carry out external validation of multicenter, large-sample, and different population-based predictive models to truly extend machine learning models into clinical practice. Furthermore, we should develop standardized operational procedures for building machine learning models, and the application of developed models in clinical practice should be regulated by authoritative organizations (eg, US Food and Drug Administration).

### Conclusion

Our analysis revealed limitations in the performance of current tools for predicting HT in patients with AIS, highlighting the lack of a well-established clinical prediction model. However, the meta-analysis demonstrated promising results for machine learning models, particularly those combining clinical features and radiomics. These findings provide encouraging evidence supporting the development and improvement of clinical prediction tools based on machine learning approaches.

## Appendix

Multimedia Appendix 1 PRISMA checklist.

Multimedia Appendix 2 Literature search strategy.

Multimedia Appendix 3 Basic characteristics of the included studies.

Multimedia Appendix 4 The result of PROBAST (Prediction Model Risk of Bias Assessment Tool) assessment.

Multimedia Appendix 5 The c-index, sensitivity, and specificity of each included model.

## Abbreviations

AIS: acute ischemic stroke
CT: computed tomography
ECASS: European Cooperative Acute Stroke Study
GRASPS: Glucose, Race, Age, Sex, systolic blood Pressure at presentation, and Severity of stroke at presentation
HBC: Heidelberg Bleeding Classification
HT: hemorrhagic transformation
MeSH: Medical Subject Headings
MSS: Multicenter rt-PA Stroke Survey score
NIH: National Institutes of Health
NIHSS: National Institutes of Health Stroke Scale
PRISMA: Preferred Reporting Items for Systematic Reviews and Meta-Analyses
PROBAST: Prediction Model Risk of Bias Assessment Tool
PROSPERO: International Prospective Register of Systematic Reviews
SEDAN: baseline blood Sugar, Early infarct signs, hyper Dense cerebral artery sign, Age, NIH Stroke Scale
sICH: symptomatic intracerebral hemorrhage
SITS: Safe Implementation of Treatments in Stroke
SPAN-100: Stroke Prognostication using Age and NIH Stroke Scale
TAG: thrombolysis in cerebral ischemia-Alberta stroke program early CT-glucose score

## Data Availability

The datasets generated or analyzed during this study are available from the corresponding author on reasonable request.
