# Supplementary material for: Early Predictive Accuracy of Machine Learning for Hemorrhagic Transformation in Acute Ischemic Stroke: Systematic Review and Meta-Analysis
Source: J Med Internet Res. 2025 May 23;27:e71654. doi: 10.2196/71654 (PMC12144484; doi:10.2196/71654)
Supplement: Multimedia Appendix 2 [file jmir_v27i1e71654_app2.docx]

**Table S1** Literature search strategy

All the searches above were up to March 2025.

**1.Pubmed**

| Search number | Query | Results |
| --- | --- | --- |
| #1 | "Stroke"[Mesh] | 189679 |
| #2 | (((((((((((((((stroke[Title/Abstract]) OR (strokes[Title/Abstract])) OR (cerebrum vascular accident[Title/Abstract])) OR (cerebrovascular accidents[Title/Abstract])) OR (cerebrovascular accident[Title/Abstract])) OR (cerebral vascular accidents[Title/Abstract])) OR (cerebral vascular accident[Title/Abstract])) OR (brain vascular accidents[Title/Abstract])) OR (brain vascular accident[Title/Abstract])) OR (brain infarcts[Title/Abstract])) OR (brain infarction[Title/Abstract])) OR (brain infarct[Title/Abstract])) OR (brain attack[Title/Abstract])) OR (brain accident[Title/Abstract])) OR (apoplexy[Title/Abstract])) OR (apoplexia[Title/Abstract]) | 373889 |
| #3 | ("Stroke"[Mesh]) OR ((((((((((((((((stroke[Title/Abstract]) OR (strokes[Title/Abstract])) OR (cerebrum vascular accident[Title/Abstract])) OR (cerebrovascular accidents[Title/Abstract])) OR (cerebrovascular accident[Title/Abstract])) OR (cerebral vascular accidents[Title/Abstract])) OR (cerebral vascular accident[Title/Abstract])) OR (brain vascular accidents[Title/Abstract])) OR (brain vascular accident[Title/Abstract])) OR (brain infarcts[Title/Abstract])) OR (brain infarction[Title/Abstract])) OR (brain infarct[Title/Abstract])) OR (brain attack[Title/Abstract])) OR (brain accident[Title/Abstract])) OR (apoplexy[Title/Abstract])) OR (apoplexia[Title/Abstract])) | 411442 |
| #4 | machine learning[MeSH Terms] | 88764 |
| #5 | (((((((((((((((((((((((machine learning[Title/Abstract]) OR (XGBoost[Title/Abstract])) OR (transfer Learning[Title/Abstract])) OR (support Vector Machine[Title/Abstract])) OR (Risk model[Title/Abstract])) OR (ResNet-50[Title/Abstract])) OR (ResNet[Title/Abstract])) OR (random forest[Title/Abstract])) OR (Radiomics[Title/Abstract])) OR (Radiomic[Title/Abstract])) OR (Prediction model[Title/Abstract])) OR (Nomogram[Title/Abstract])) OR (neural networks[Title/Abstract])) OR (neural network[Title/Abstract])) OR (Naive Bayesian[Title/Abstract])) OR (Multilayer perceptron[Title/Abstract])) OR (K-Nearest Neighbor[Title/Abstract])) OR (Gradient Boosting Machine[Title/Abstract])) OR (Ensemble Learning[Title/Abstract])) OR (deep Learning[Title/Abstract])) OR (Decision tree[Title/Abstract])) OR (Bayesian network[Title/Abstract])) OR (artificial intelligence[Title/Abstract])) OR (Adaboost[Title/Abstract]) | 421544 |
| #6 | "machine learning"[MeSH Terms] OR "machine learning"[Title/Abstract] OR "XGBoost"[Title/Abstract] OR "transfer learning"[Title/Abstract] OR "support vector machine"[Title/Abstract] OR "risk model"[Title/Abstract] OR "ResNet-50"[Title/Abstract] OR "ResNet"[Title/Abstract] OR "random forest"[Title/Abstract] OR "Radiomics"[Title/Abstract] OR "Radiomic"[Title/Abstract] OR "prediction model"[Title/Abstract] OR "Nomogram"[Title/Abstract] OR "neural networks"[Title/Abstract] OR "neural network"[Title/Abstract] OR "naive bayesian"[Title/Abstract] OR "multilayer perceptron"[Title/Abstract] OR "k nearest neighbor"[Title/Abstract] OR "gradient boosting machine"[Title/Abstract] OR "ensemble learning"[Title/Abstract] OR "deep learning"[Title/Abstract] OR "decision tree"[Title/Abstract] OR "bayesian network"[Title/Abstract] OR "artificial intelligence"[Title/Abstract] OR "Adaboost"[Title/Abstract] | 428570 |
| #7 | intracranial hemorrhages[MeSH Terms] | 84421 |
| #8 | ((((((((((((((((((((intracranial hemorrhages[Title/Abstract]) OR (Posterior Fossa Hemorrhages[Title/Abstract])) OR (Posterior Fossa Hemorrhage[Title/Abstract])) OR (intracranial hemorrhages[Title/Abstract])) OR (intracranial hemorrhage[Title/Abstract])) OR (intracranial haemorrhages[Title/Abstract])) OR (intracranial haemorrhage[Title/Abstract])) OR (intracranial bleeding[Title/Abstract])) OR (intracerebral hemorrhage[Title/Abstract])) OR (intracerebral haemorrhage[Title/Abstract])) OR (intracerebral bleeding[Title/Abstract])) OR (hemorrhagic transformations[Title/Abstract])) OR (hemorrhagic transformation[Title/Abstract])) OR (hematencephalon[Title/Abstract])) OR (encephalorrhagia[Title/Abstract])) OR (cerebral hemorrhage[Title/Abstract])) OR (cerebral haemorrhage[Title/Abstract])) OR (brain hemorrhages[Title/Abstract])) OR (brain hemorrhage[Title/Abstract])) OR (brain haemorrhage[Title/Abstract])) OR (brain bleeding[Title/Abstract]) | 51472 |
| #9 | "intracranial hemorrhages"[MeSH Terms] OR "intracranial hemorrhages"[Title/Abstract] OR "posterior fossa hemorrhages"[Title/Abstract] OR "posterior fossa hemorrhage"[Title/Abstract] OR "intracranial hemorrhages"[Title/Abstract] OR "intracranial hemorrhage"[Title/Abstract] OR "intracranial haemorrhages"[Title/Abstract] OR "intracranial haemorrhage"[Title/Abstract] OR "intracranial bleeding"[Title/Abstract] OR "intracerebral hemorrhage"[Title/Abstract] OR "intracerebral haemorrhage"[Title/Abstract] OR "intracerebral bleeding"[Title/Abstract] OR "hemorrhagic transformations"[Title/Abstract] OR "hemorrhagic transformation"[Title/Abstract] OR "hematencephalon"[Title/Abstract] OR "encephalorrhagia"[Title/Abstract] OR "cerebral hemorrhage"[Title/Abstract] OR "cerebral haemorrhage"[Title/Abstract] OR "brain hemorrhages"[Title/Abstract] OR "brain hemorrhage"[Title/Abstract] OR "brain haemorrhage"[Title/Abstract] OR "brain bleeding"[Title/Abstract] | 109666 |
| #10 | ("Stroke"[MeSH Terms] OR ("Stroke"[Title/Abstract] OR "strokes"[Title/Abstract] OR (("cerebrum"[MeSH Terms] OR "cerebrum"[All Fields] OR "cerebrums"[All Fields]) AND "vascular accident"[Title/Abstract]) OR "cerebrovascular accidents"[Title/Abstract] OR "cerebrovascular accident"[Title/Abstract] OR "cerebral vascular accidents"[Title/Abstract] OR "cerebral vascular accident"[Title/Abstract] OR "brain vascular accidents"[Title/Abstract] OR "brain vascular accident"[Title/Abstract] OR "brain infarcts"[Title/Abstract] OR "brain infarction"[Title/Abstract] OR "brain infarct"[Title/Abstract] OR "brain attack"[Title/Abstract] OR (("brain"[MeSH Terms] OR "brain"[All Fields] OR "brains"[All Fields] OR "brain s"[All Fields]) AND "accident"[Title/Abstract]) OR "apoplexy"[Title/Abstract] OR "apoplexia"[Title/Abstract])) AND ("machine learning"[MeSH Terms] OR ("machine learning"[Title/Abstract] OR "XGBoost"[Title/Abstract] OR "transfer learning"[Title/Abstract] OR "support vector machine"[Title/Abstract] OR "risk model"[Title/Abstract] OR "ResNet-50"[Title/Abstract] OR "ResNet"[Title/Abstract] OR "random forest"[Title/Abstract] OR "Radiomics"[Title/Abstract] OR "Radiomic"[Title/Abstract] OR "prediction model"[Title/Abstract] OR "Nomogram"[Title/Abstract] OR "neural networks"[Title/Abstract] OR "neural network"[Title/Abstract] OR "naive bayesian"[Title/Abstract] OR "multilayer perceptron"[Title/Abstract] OR "k nearest neighbor"[Title/Abstract] OR "gradient boosting machine"[Title/Abstract] OR "ensemble learning"[Title/Abstract] OR "deep learning"[Title/Abstract] OR "decision tree"[Title/Abstract] OR "bayesian network"[Title/Abstract] OR "artificial intelligence"[Title/Abstract] OR "Adaboost"[Title/Abstract])) AND ("intracranial hemorrhages"[MeSH Terms] OR ("intracranial hemorrhages"[Title/Abstract] OR "posterior fossa hemorrhages"[Title/Abstract] OR "posterior fossa hemorrhage"[Title/Abstract] OR "intracranial hemorrhages"[Title/Abstract] OR "intracranial hemorrhage"[Title/Abstract] OR "intracranial haemorrhages"[Title/Abstract] OR "intracranial haemorrhage"[Title/Abstract] OR "intracranial bleeding"[Title/Abstract] OR "intracerebral hemorrhage"[Title/Abstract] OR "intracerebral haemorrhage"[Title/Abstract] OR "intracerebral bleeding"[Title/Abstract] OR "hemorrhagic transformations"[Title/Abstract] OR "hemorrhagic transformation"[Title/Abstract] OR "hematencephalon"[Title/Abstract] OR "encephalorrhagia"[Title/Abstract] OR "cerebral hemorrhage"[Title/Abstract] OR "cerebral haemorrhage"[Title/Abstract] OR "brain hemorrhages"[Title/Abstract] OR "brain hemorrhage"[Title/Abstract] OR "brain haemorrhage"[Title/Abstract] OR "brain bleeding"[Title/Abstract])) | 483 |

**2.Cochrane**

| Search number | Query | Results |
| --- | --- | --- |
| #1 | MeSH descriptor: [Stroke] explode all trees | 17720 |
| #2 | (stroke):ti,ab,kw OR (strokes):ti,ab,kw OR (cerebrum vascular accident):ti,ab,kw OR (cerebrovascular accidents):ti,ab,kw OR (cerebrovascular accident):ti,ab,kw | 79064 |
| #3 | (cerebral vascular accidents):ti,ab,kw OR (cerebral vascular accident):ti,ab,kw OR (brain vascular accidents):ti,ab,kw OR (brain vascular accident):ti,ab,kw OR (brain infarcts):ti,ab,kw | 1074 |
| #4 | (brain infarction):ti,ab,kw OR (brain infarct):ti,ab,kw OR (brain attack):ti,ab,kw OR (brain accident):ti,ab,kw OR (apoplexy):ti,ab,kw | 10191 |
| #5 | (apoplexia):ti,ab,kw | 7 |
| #6 | #1 OR #2 OR #3 OR #4 OR #5 | 81984 |
| #7 | MeSH descriptor: [Machine Learning] explode all trees | 1091 |
| #8 | (machine learning):ti,ab,kw OR (XGBoost):ti,ab,kw OR (transfer Learning):ti,ab,kw OR (support Vector Machine):ti,ab,kw OR (Risk model):ti,ab,kw | 37568 |
| #9 | (ResNet-50):ti,ab,kw OR (ResNet):ti,ab,kw OR (random forest):ti,ab,kw OR (Radiomics):ti,ab,kw OR (Radiomic):ti,ab,kw | 1751 |
| #10 | (Prediction model):ti,ab,kw OR (Nomogram):ti,ab,kw OR (neural networks):ti,ab,kw OR (neural network):ti,ab,kw OR (Naive Bayesian):ti,ab,kw | 11033 |
| #11 | (Multilayer perceptron):ti,ab,kw OR (K-Nearest Neighbor):ti,ab,kw OR (Gradient Boosting Machine):ti,ab,kw OR (Ensemble Learning):ti,ab,kw OR (deep Learning):ti,ab,kw | 2372 |
| #12 | (Decision tree):ti,ab,kw OR (Bayesian network):ti,ab,kw OR (artificial intelligence):ti,ab,kw OR (Adaboost):ti,ab,kw | 4093 |
| #13 | #7 OR #8 OR #9 OR #10 OR #11 OR #12 | 48414 |
| #14 | MeSH descriptor: [Intracranial Hemorrhages] explode all trees | 3202 |
| #15 | (intracranial hemorrhages):ti,ab,kw OR (Posterior Fossa Hemorrhages):ti,ab,kw OR (Posterior Fossa Hemorrhage):ti,ab,kw OR (intracranial hemorrhages):ti,ab,kw OR (intracranial hemorrhage):ti,ab,kw | 4359 |
| #16 | (intracranial haemorrhages):ti,ab,kw OR (intracranial haemorrhage):ti,ab,kw OR (intracranial bleeding):ti,ab,kw OR (intracerebral hemorrhage):ti,ab,kw OR (intracerebral haemorrhage):ti,ab,kw | 7397 |
| #17 | (intracerebral bleeding):ti,ab,kw OR (hemorrhagic transformations):ti,ab,kw OR (hemorrhagic transformation):ti,ab,kw OR (hematencephalon):ti,ab,kw OR (encephalorrhagia):ti,ab,kw | 712 |
| #18 | (cerebral hemorrhage):ti,ab,kw OR (cerebral haemorrhage):ti,ab,kw OR (brain hemorrhages):ti,ab,kw OR (brain hemorrhage):ti,ab,kw OR (brain haemorrhage):ti,ab,kw | 10908 |
| #19 | (brain bleeding):ti,ab,kw | 3061 |
| #20 | #14 OR #15 OR #16 OR #17 OR #18 OR #19 | 14429 |
| #21 | #6 AND #13 AND #20 | 388 |

**3.Embase**

| Search number | Query | Results |
| --- | --- | --- |
| #1 | 'cerebrovascular accident'/exp | 486929 |
| #2 | 'stroke':ab,ti OR 'strokes':ab,ti OR 'cerebrum vascular accident':ab,ti OR 'cerebrovascular accidents':ab,ti OR 'cerebrovascular accident':ab,ti OR 'cerebral vascular accidents':ab,ti OR 'cerebral vascular accident':ab,ti OR 'brain vascular accidents':ab,ti OR 'brain vascular accident':ab,ti OR 'brain infarcts':ab,ti OR 'brain infarction':ab,ti OR 'brain infarct':ab,ti OR 'brain attack':ab,ti OR 'brain accident':ab,ti OR apoplexy:ab,ti OR apoplexia:ab,ti | 574071 |
| #3 | #1 OR #2 | 697158 |
| #4 | 'machine learning'/exp | 567264 |
| #5 | 'machine learning':ab,ti OR xgboost:ab,ti OR 'transfer learning':ab,ti OR 'support vector machine':ab,ti OR 'risk model':ab,ti OR 'resnet 50':ab,ti OR resnet:ab,ti OR 'random forest':ab,ti OR radiomics:ab,ti OR radiomic:ab,ti OR 'prediction model':ab,ti OR nomogram:ab,ti OR 'neural networks':ab,ti OR 'neural network':ab,ti OR 'naive bayesian':ab,ti OR 'multilayer perceptron':ab,ti OR 'k-nearest neighbor':ab,ti OR 'gradient boosting machine':ab,ti OR 'ensemble learning':ab,ti OR 'deep learning':ab,ti OR 'decision tree':ab,ti OR 'bayesian network':ab,ti OR 'artificial intelligence':ab,ti OR adaboost:ab,ti | 475046 |
| #6 | #4 OR #5 | 751138 |
| #7 | 'brain hemorrhage'/exp | 203856 |
| #8 | 'posterior fossa hemorrhages':ab,ti OR 'posterior fossa hemorrhage':ab,ti OR 'intracranial hemorrhages':ab,ti OR 'intracranial hemorrhage':ab,ti OR 'intracranial haemorrhages':ab,ti OR 'intracranial haemorrhage':ab,ti OR 'intracranial bleeding':ab,ti OR 'intracerebral hemorrhage':ab,ti OR 'intracerebral haemorrhage':ab,ti OR 'intracerebral bleeding':ab,ti OR 'hemorrhagic transformations':ab,ti OR 'hemorrhagic transformation':ab,ti OR hematencephalon:ab,ti OR encephalorrhagia:ab,ti OR 'cerebral hemorrhage':ab,ti OR 'cerebral haemorrhage':ab,ti OR 'brain hemorrhages':ab,ti OR 'brain hemorrhage':ab,ti OR 'brain haemorrhage':ab,ti OR 'brain bleeding':ab,ti | 75474 |
| #9 | #7 OR #8 | 215812 |
| #10 | #3 AND #6 AND #9 | 1309 |

**4.Web of science**

| Search number | Query | Results |
| --- | --- | --- |
| #1 | stroke (Topic) OR strokes (Topic) OR cerebrum vascular accident (Topic) OR cerebrovascular accidents (Topic) OR cerebrovascular accident (Topic) OR cerebral vascular accidents (Topic) OR cerebral vascular accident (Topic) OR brain vascular accidents (Topic) OR brain vascular accident (Topic) OR brain infarcts (Topic) OR brain infarction (Topic) OR brain infarct (Topic) OR brain attack (Topic) OR brain accident (Topic) OR apoplexy (Topic) OR apoplexia (Topic) | 441004 |
| #2 | machine learning (Topic) OR XGBoost (Topic) OR transfer Learning (Topic) OR support Vector Machine (Topic) OR Risk model (Topic) OR ResNet-50 (Topic) OR ResNet (Topic) OR random forest (Topic) OR Radiomics (Topic) OR Radiomic (Topic) OR Prediction model (Topic) OR Nomogram (Topic) OR neural networks (Topic) OR neural network (Topic) OR Naive Bayesian (Topic) OR Multilayer perceptron (Topic) OR K-Nearest Neighbor (Topic) OR Gradient Boosting Machine (Topic) OR Ensemble Learning (Topic) OR deep Learning (Topic) OR Decision tree (Topic) OR Bayesian network (Topic) OR artificial intelligence (Topic) OR Adaboost (Topic) | 2589566 |
| #3 | intracranial hemorrhages (Topic) OR Posterior Fossa Hemorrhages (Topic) OR Posterior Fossa Hemorrhage (Topic) OR intracranial hemorrhages (Topic) OR intracranial hemorrhage (Topic) OR intracranial haemorrhages (Topic) OR intracranial haemorrhage (Topic) OR intracranial bleeding (Topic) OR intracerebral hemorrhage (Topic) OR intracerebral haemorrhage (Topic) OR intracerebral bleeding (Topic) OR hemorrhagic transformations (Topic) OR hemorrhagic transformation (Topic) OR hematencephalon (Topic) OR encephalorrhagia (Topic) OR cerebral hemorrhage (Topic) OR cerebral haemorrhage (Topic) OR brain hemorrhages (Topic) OR brain hemorrhage (Topic) OR brain haemorrhage (Topic) OR brain bleeding (Topic) | 88803 |
| #4 | #1 AND #2 AND #3 | 4048 |
